# High-Density SNP Mapping of the HLA Region Identifies Multiple Independent Susceptibility Loci Associated with Selective IgA Deficiency

**DOI:** 10.1371/journal.pgen.1002476

**Published:** 2012-01-26

**Authors:** Ricardo C. Ferreira, Qiang Pan-Hammarström, Robert R. Graham, Gumersindo Fontán, Annette T. Lee, Ward Ortmann, Ning Wang, Elena Urcelay, Miguel Fernández-Arquero, Concepción Núñez, Gudmundur Jorgensen, Björn R. Ludviksson, Sinikka Koskinen, Katri Haimila, Leonid Padyukov, Peter K. Gregersen, Lennart Hammarström, Timothy W. Behrens

**Affiliations:** 1 Genentech, South San Francisco, California, United States of America; 2 Division of Clinical Immunology, Department of Laboratory Medicine, Karolinska Institutet at Karolinska University Hospital, Huddinge, Stockholm, Sweden; 3 Department of Immunology, Hospital Universitario La Paz, Madrid, Spain; 4 The Feinstein Institute for Medical Research, North Shore-Long Island Jewish Health System, Manhasset, New York, United States of America; 5 Department of Clinical Immunology, Hospital Clínico San Carlos, Madrid, Spain; 6 Landspitali–University Hospital and the Department of Medicine, University of Iceland, Reykjavik, Iceland; 7 Finnish Red Cross Blood Service, Clinical Laboratory, Helsinki, Finland; 8 Rheumatology Unit, Department of Medicine, Karolinska Institutet, Stockholm, Sweden; University of Utah School of Medicine, United States of America

## Abstract

Selective IgA deficiency (IgAD; serum IgA<0.07 g/l) is the most common form of human primary immune deficiency, affecting approximately 1∶600 individuals in populations of Northern European ancestry. The polygenic nature of IgAD is underscored by the recent identification of several new risk genes in a genome-wide association study. Among the characterized susceptibility loci, the association with specific HLA haplotypes represents the major genetic risk factor for IgAD. Despite the robust association, the nature and location of the causal variants in the HLA region remains unknown. To better characterize the association signal in this region, we performed a high-density SNP mapping of the HLA locus and imputed the genotypes of common *HLA-B*, *-DRB1*, and -*DQB1* alleles in a combined sample of 772 IgAD patients and 1,976 matched controls from 3 independent European populations. We confirmed the complex nature of the association with the HLA locus, which is the result of multiple effects spanning the entire HLA region. The primary association signal mapped to the HLA-DQB1*02 allele in the HLA Class II region (combined *P* = 7.69×10^−57^; OR = 2.80) resulting from the combined independent effects of the HLA-B*0801-DRB1*0301-DQB1*02 and -DRB1*0701-DQB1*02 haplotypes, while additional secondary signals were associated with the DRB1*0102 (combined *P* = 5.86×10^−17^; OR = 4.28) and the DRB1*1501 (combined *P* = 2.24×10^−35^; OR = 0.13) alleles. Despite the strong population-specific frequencies of HLA alleles, we found a remarkable conservation of these effects regardless of the ethnic background, which supports the use of large multi-ethnic populations to characterize shared genetic association signals in the HLA region. We also provide evidence for the location of association signals within the specific extended haplotypes, which will guide future sequencing studies aimed at characterizing the precise functional variants contributing to disease pathogenesis.

## Author Summary

The human leukocyte antigen (HLA) locus is robustly associated with many immune-mediated conditions. However, identification of the genetic variants contributing to the disease pathophysiology has been greatly hampered by the extensive chromosomal conservation within this genomic region. To better understand the association of the HLA locus in selective IgA deficiency (IgAD), we used an extensive genotyping database from a recent genome-wide association study (GWAS) to generate a high-density SNP map of this region in a combined sample of >2,700 individuals from 3 independent European populations. In addition, we took advantage of recent methodological advances to impute the more common *HLA-B*, *-DRB1*, and *-DQB1* alleles in all subjects. We confirmed the strong disease-association of the HLA locus and identified several different signals located in specific conserved HLA haplotypes contributing independent risk or protection for IgAD. Further analysis of the chromosomal sequences associated with the associated HLA alleles allowed us to refine the mapping of the susceptibility variants. These findings represent the most comprehensive high-density SNP mapping of the HLA locus in IgAD to date and provide important new information as to the location of the genetic variants contributing to this common immune deficiency.

## Introduction

The major histocompatibility complex (MHC) locus has been one of the most intensively studied regions in the vertebrate genome since it was first discovered in the mouse in 1936 [1]. In humans, gene products from the MHC were initially identified as surface markers on leucocytes, which led to its alternative designation as the human leukocyte antigen (HLA) complex. This genomic region spans approximately 3.6 megabase pairs (Mb) of genomic sequence and encodes over 200 genes, many of which with a defined immune function [2]. More recently, the definition of an extended MHC region (xMHC) has been proposed, which encompasses approximately 7.6 Mb and 421 annotated gene loci [3]. The HLA locus contains the canonical HLA Class I and Class II gene clusters. Class I HLA molecules are expressed on the surface of most human cells, while constitutive expression of Class II molecules is restricted to antigen presenting cells, including dendritic cells, macrophages and B cells. These molecules function to present peptide antigens to T cells to initiate adaptive immune responses.

The relevance of this locus to the pathogenesis of common human diseases is clearly evidenced by the reported association of polymorphisms in the HLA region with over one hundred diseases, particularly autoimmune and inflammatory conditions [4]. Similarly, the genetic association with HLA markers has been well documented in selective IgA deficiency (IgAD; serum IgA concentration <0.07 g/l), the most common form of human primary immunodeficiency [5], [6]. However, despite the robust association with a large number of immune-mediated conditions, the conservation of some ancestral haplotypes [7], [8], which can encompass large genomic segments containing many genes, has greatly impaired our ability to unambiguously identify the gene(s) contributing to disease susceptibility.

The HLA was first described as a risk locus for IgAD through the association with HLA Class I and Class II markers [9]–[11] and ascribed to specific conserved haplotypes. Most notably, the extended HLA-A*01-B*08-DRB1*0301-DQB1*02 (DR3) haplotype has been identified as the single strongest genetic risk factor for IgAD in Northern European populations [12]. A striking 13% of DR3 homozygotes have been estimated to be IgA deficient [13], although this figure might be inflated due to publication bias [14]. An even larger proportion (67%) appears to suffer from at least one form of Ig deficiency, including selective IgG3, IgG4, IgD and IgA deficiency [15]. Conversely, the HLA-DRB1*1501-DQB1*06 (DR2) haplotype, has been shown to confer strong protection against IgAD, with homozygous individuals showing a virtual complete protection from the disease [12]. Positive associations have also been described with 2 other extended haplotypes, namely the HLA-B*14-DRB1*0102-DQB1*05 (DR1) and the HLA-B*44-DRB1*0701-DQB1*02 (DR7) haplotypes [12]. Interestingly, the risk conferred by DR7 and particularly DR1 haplotypes has been shown to be greater than that conferred by DR3 haplotypes in populations of Southern European ancestry [16]–[18]. Despite the strong association at the HLA locus, there has been no consensus as to the precise location of the causal variants within these haplotypes, with some groups suggesting its placement to the telomeric end of the HLA Class III region [19]–[21] and others in the HLA Class II region [22], [23].

To refine the complex association signals in the extended MHC, we genotyped a panel of 1,686 SNPs and imputed with high confidence the genotype of 9,905 SNPs spanning the entire HLA locus. We used the genotype information to reconstruct the individual long-range haplotypes and impute common HLA alleles. Here, we report single and multi-marker association analyses of both HLA and non-HLA variants with IgAD in a combined sample of 772 IgAD cases and 1976 matched controls from 3 independent European populations. These data provide the most complete fine-mapping effort of the HLA locus in IgAD to date.

## Results

### Association of HLA and non-HLA common variants using a high-density SNP–based mapping approach

We recently performed a genome-wide association study of IgAD and reported the disease-association of 2 novel non-HLA loci: *IFIH1* and *CLEC16A* [24]. We also confirmed the HLA locus as the strongest genetic risk factor for IgAD [24]. To better characterize the association at the HLA locus, we used the genotyping data from 1,686 SNPs located within a 10 Mb region of chromosome 6 (25–35 Mb) containing the extended HLA region, and employed a long-range haplotype phasing approach to infer, with high confidence, individual HLA haplotypes. To further refine the fine mapping of this region, we also imputed the genotypes of an additional 9,905 SNPs using the HapMap2 reference set.

Allele-based association tests were then performed on the 1,686 genotyped and 9,905 imputed SNPs and on the imputed HLA alleles that survived stringent quality control steps in 430 IgAD cases and 1090 controls from Sweden and Iceland and in two independent replication cohorts from Spain (256 cases and 322 controls) and Finland (86 cases and 564 controls). To avoid spurious associations due to specific population differences that are associated with the HLA locus, we used all available genome-wide genotyping data to describe the population substructure using a principal component method [25], [26]. Exclusion of the genetic outliers in each population ensured the genetic homogeneity of our sample, as evidenced by the modest distributional inflation (λ_GC_) observed in each case-control series (Sweden/Iceland: λ_GC_ = 1.05; Spain: λ_GC_ = 1.05; Finland: λ_GC_ = 1.04). Allele-based association tests were performed in each population independently, and combined *P* values were calculated using a meta-analysis. In addition, we also phased the genotypes from a set of 49 selected SNPs spanning the classical MHC region to reconstruct the extended haplotypes in each individual. SNP selection was optimized to combine a set of 11 SNPs that best captured the information of the known HLA alleles that were typed directly in our sample, together with a second set of 38 common SNPs, which had low pairwise LD and were distributed uniformly across the classical HLA region. Importantly, this strategy ensured that we recapitulated the full diversity of the haplotype structure of the HLA locus, and allowed us to map the individual recombination events within the extended haplotypes (see Methods). We tested the association of the more common extended haplotypes and used the haplotype background information to map the recombination events more accurately and the location of the putative causal variants within each specific disease-associated haplotype.

### Additive effect of two independent risk factors in the HLA-B*0801-DRB1*0301 and HLA-DRB1*0701-DQB1*02 haplotypes is responsible for the primary association signal in the HLA Class II region

**Figure 1:**
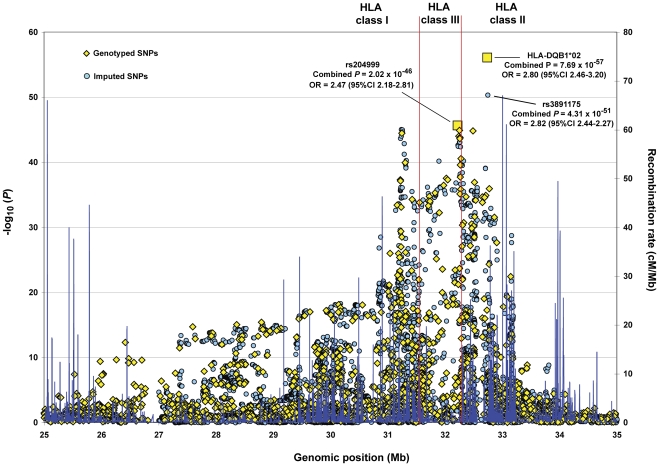
Primary association signal of the HLA locus with IgAD. Allele-based association tests were performed for all 1,686 genotyped SNPs and imputed HLA alleles (represented by yellow diamonds) and for the 9,905 SNPs imputed with high confidence using the HapMap2 reference set (represented by light blue circles). All association results are represented as the −log_10_ of the combined *P* values (left y-axis). The most associated SNP and HLA allele are indicated by yellow squares. Recombination rates from the HapMap CEU are depicted in dark blue (right y-axis). Genomic positions on the x-axis are based on the NCBI Build 36 (hg 18) assembly. CI, confidence interval; Mb, megabase pairs.

The primary association peak in the HLA locus mapped to the Class II region (Figure 1). Consistent with a recent report, that included approximately 270 overlapping IgAD patients and 670 controls from the Swedish cohort used in this study [27], the strongest association was observed with the imputed HLA-DQB1*02 allele (combined *P* = 7.69×10^−57^; OR = 2.80, 95% CI 2.46–3.20; Figure 1). The association with the DQB1*02 allele was approximately 6 orders of magnitude more significant than the most associated imputed SNP, which mapped to the HLA-DRB1 gene (rs3891175; combined *P* = 4.31×10^−51^; OR = 2.82, 95% CI 2.44-2.27) and over 10^10^-fold more significant than the most associated genotyped marker (rs204999; combined *P* = 2.02×10^−46^; OR = 2.47, 95% CI 2.18–2.81; Figure 1).

HLA-DQB1*02 is a common allele in populations of European ancestry, and is linked to both the HLA-DRB1*0301 and -DRB1*0701 alleles that have been previously described as strong IgAD risk factors. In this study, we confirmed the disease risk conferred by the DRB1*0301 (combined *P* = 1.56×10^−34^; OR = 2.49, 95% CI 2.14–2.90; Table 1) and DRB1*0701 alleles (combined *P* = 8.68×10^−17^; OR = 2.03, 95% CI 1.70–2.41; Table 1). One important distinction between these 2 haplotypes is that the DRB1*0301 allele is typically linked with the DQB1*0201 allele, while the DRB1*0701 allele is linked to the DQB1*0202 variant [28]. These two DQB1*02 alleles differ only by one amino acid in the third exon, and are therefore difficult to differentiate by traditional typing methods using 2-digit resolution. Despite the sequence similarity, the HLA Class II sequences surrounding these 2 alleles are distinct and contain different *HLA-DQA1* alleles. To discriminate between the two HLA-DQB1*02 alleles, we genotyped the HLA-DQB1 gene in 15 Finnish IgAD patients carrying the DQB1*02 allele (5 DRB1*0301/X, 5 DRB1*0701/X and 5 DRB1*0301/DRB1*0701 heterozygous individuals) using 4-digit resolution. We confirmed that all DRB1*0301 alleles were always associated with the DQB1*0201 allele, while 9/10 (90%) of the DRB1*0701 alleles were associated with the DQB1*0202 subtype (data not shown).

**Table 1:** Association of common HLA haplotypes with IgAD.

		Sweden/Iceland (430 Cases, 1090 Controls)				Spain (256 Cases, 322 Controls)				Finland (86 Cases, 564 Controls)					
HLA haplotype	N (%)	Freq Cases	Freq Controls	*P* value	OR	Freq Cases	Freq Controls	*P* value	OR	Freq Cases	Freq Controls	*P* value	OR	Combined *P* value	Combined OR (95% CI)
DRB1*0301	907	0.295	0.136	2.87×10^−21^	2.54	0.205	0.120	7.37×10^−5^	1.96	0.273	0.114	1.75×10^−7^	2.78	1.56×10^−34^	2.49 (2.14–2.90)
B*0801-DRB1*0301-DQB1*02	634 (69.9%)	0.254	0.097	9.79×10^−24^	2.96	0.106	0.026	2.26×10^−7^	4.50	0.233	0.083	1.80×10^−8^	3.57	3.37×10^−43^	3.33 (2.79–3.97)
Recombinant DRB1*0301 (non-B*0801)	273 (30.1%)	0.042	0.039	0.712	1.08	0.100	0.093	0.708	1.08	0.041	0.03	0.455	1.38	0.424	1.10 (0.84–1.44)
DRB1*0701	625	0.145	0.081	1.91×10^−7^	1.93	0.277	0.165	6.76×10^−6^	1.94	0.134	0.046	3.66×10^−5^	2.94	8.68×10^−17^	2.03 (1.70–2.41)
B*1302-DRB1*0701-DQB1*02	90 (14.4%)	0.017	0.008	0.037	2.06	0.023	0.009	0.061	2.59	0.076	0.023	3.13×10^−4^	3.69	1.36×10^−5^	2.63 (1.69–4.09)
Recombinant DRB1*0701-DQB1*02 (non-B*1302)	463 (74.1%)	0.109	0.049	3.42×10^−9^	2.44	0.229	0.132	3.57×10^−5^	1.92	0.105	0.038	3.13×10^−4^	2.92	6.35×10^−17^	2.23 (1.83–2.72)
Recombinant DRB1*0701 (non-DQB1*02)	162 (25.9%)	0.036	0.033	0.629	1.11	0.049	0.033	0.165	1.52	0.029	0.008	0.019	3.81	0.027	1.34 (0.96–1.86)
DRB1*0102	140	0.027	0.003	6.42×10^−7^	8.74	0.141	0.045	3.36×10^−8^	3.69	0.023	0.004	0.013	5.45	5.86×10^−17^	4.28 (2.92–6.26)
B*1402-DRB1*0102-DQB1*05	93 (66.4%)	0.023	0.002	2.72×10^−6^	13.24	0.086	0.030	6.77×10^−5^	3.11	0.023	0.002	2.74×10^−3^	13.71	1.24×10^−16^	4.59 (2.89–7.30)
Recombinant DRB1*0102 (non-B*1402)	47 (33.6%)	0.003	0.001	0.254	2.55	0.055	0.016	3.88×10^−4^	3.83	0	0.003	0.498	N/A	3.62×10^−3^	3.22 (1.70–6.13)
DRB1*0101-DQB1*05	786	0.123	0.121	0.848	1.02	0.102	0.107	0.762	0.94	0.337	0.211	4.20×10^−4^	1.86	0.038	1.17 (0.98–1.39)
DRB1*1501	*574*	0.027	0.151	9.82×10^−17^	0.16	0.008	0.107	5.08×10^−7^	0.07	0.017	0.13	2.65×10^−4^	0.12	2.24×10^−35^	0.13 (0.09–0.19)
B*0702-DRB1*1501-DQB1*06	280 (48.8%)	0.015	0.077	5.90×10^−9^	0.18	0.002	0.034	5.65×10^−3^	0.06	0	0.067	4.51×10^−4^	N/A	8.23×10^−17^	0.14 (0.08–0.24)
Recombinant DRB1*15 (non-B*0702)	294 (51.2%)	0.012	0.073	1.28×10^−8^	0.15	0.006	0.073	1.77×10^−5^	0.08	0.017	0.063	0.021	0.25	3.70×10^−18^	0.14 (0.09–0.24)

**Figure 2:**
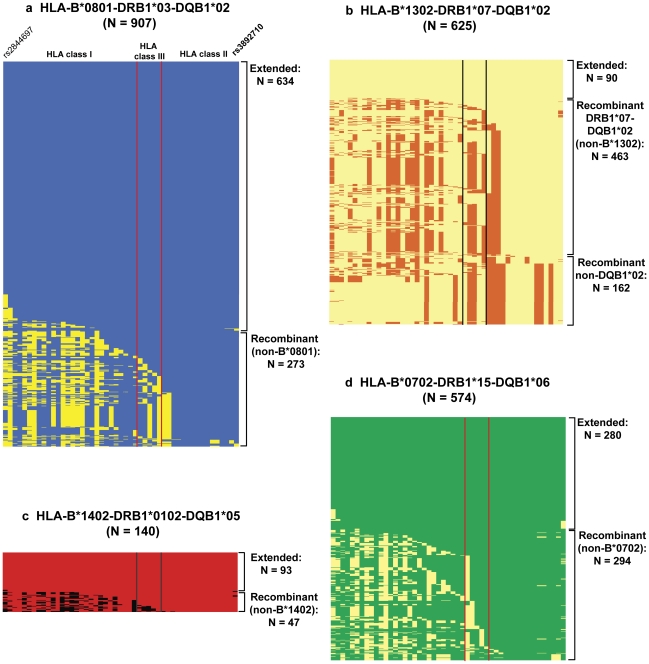
Representation of the haplotypes carrying the *HLA-DRB1* alleles most associated with IgAD. Reconstructed extended haplotypes using the final set of 49 SNPs spanning the classical HLA region were aligned to the respective extended haplotype. Shown here are the reconstructed haplotypes carrying the HLA-DRB1*03 (a), -DRB1*07 (b), -DRB1*0102 (c) and -DRB1*15 (d) alleles. Recombinant haplotypes represent all haplotypes containing the same *HLA-DRB1* allele in association with a different *HLA-B* allele, as compared to the respective extended haplotype. Vertical bars delimit the HLA Class III region. N, Number of haplotypes. Haplotype frequencies in the Swedish/Icelandic, Spanish and Icelandic cohorts are detailed in Table 1.

Shown are the summary statistics describing the association of select haplotypes on the 3 independent European case-control series. Recombinant haplotypes were defined as segments of the respective extended haplotypes and result from the occurrence of a recombination event between the HLA-B and HLA-DRB1 genes. Combined *P* values and odds ratios were calculated as described in Methods. N, number of haplotypes; Freq, frequency; OR, odds ratio; N/A, not available. Extended haplotypes and individual recombination events are depicted in Figure 2.

Analysis of the haplotype structure showed that, as noted previously [29], DRB1*0301 haplotypes showed very low levels of historical recombination and were generally found as extended conserved haplotypes (Figure 2). In our sample, of the 907 haplotypes carrying the DRB1*0301 allele, 634 (69.9%) encompassed the extended HLA-B*0801-DRB1*0301-DQB1*02 haplotype, and 273 (30.1%) were recombinant (non-B*0801) haplotypes. Importantly, recombinant DRB1*0301 haplotypes that lacked B*0801 showed no evidence of association with IgAD (combined *P* = 0.42; OR = 1.10, 95% CI 0.84–1.44; Table 1). In contrast, the extended HLA-B*0801-DRB1*0301-DQB1*02 haplotype was found to be the most disease-associated HLA haplotype (combined *P* = 3.37×10^−43^; OR = 3.33, 95% CI 2.79–3.97; Table 1).

The distribution of DRB1*03 haplotypes varied across the populations studied, with a higher percentage of extended HLA-B*0801-DRB1*0301-DQB1*02 haplotypes in Northern Europeans, as compared with the Spanish population (Table S1), likely reflecting the increased frequency of HLA-B*18-DRB1*0301-DQB1*02 haplotypes in Southern Europeans. Nevertheless, in all 3 cohorts, we observed a consistent and significant increase in the frequency of extended B*0801-DRB1*0301-DQB1*02 compared to recombinant (non-B*0801) DRB1*0301 haplotypes in IgAD cases (combined *P* = 6.3×10^−9^; OR = 2.65, 95% CI 1.90–3.71; Table S1). Similarly, recombinant (non-DRB1*0301) B*0801 haplotypes were also not associated with IgAD (combined *P* = 0.228; OR = 0.92, 95% CI 0.64–1.33; data not shown). Given the strong effect size of the risk allele present on the extended HLA-B*0801-DRB1*0301-DQB1*02 haplotype, we have 100% and 96% power to detect the association with the observed recombinant (non-B*0801) DRB1*0301 and (non-DRB1*0301) B*0801 haplotypes, respectively, at a significance level (α) of 5×10^−5^, and even 100% and 50% power at a much more stringent genome-wide significant threshold of 5×10^−8^ (Table S2).

**Figure 3:**
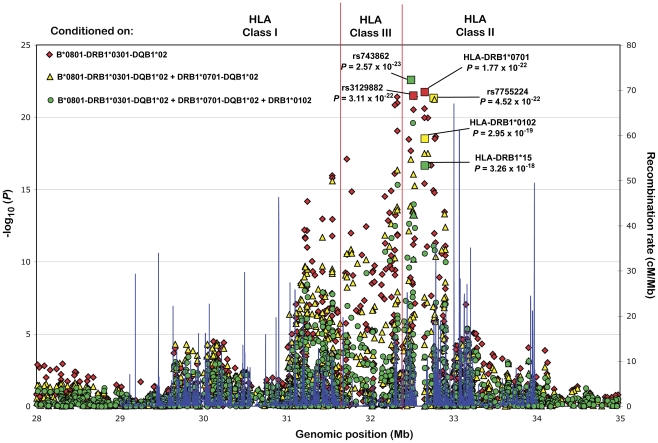
Secondary association signals in the HLA locus. Combined *P* values are shown for all SNPs and imputed HLA alleles, following conditioning on the HLA-B*0801-DRB1*0301-DQB1*02 (red diamonds), the HLA-B*0801-DRB1*0301-DQB1*02+HLA-DRB1*0701-DQB1*02 (yellow triangles) and the HLA-B*0801-DRB1*0301-DQB1*02+HLA-DRB1*0701-DQB1*02+HLA-DRB1*0102 (green circles) haplotypes. All association results are represented as the −log_10_ of the combined *P* values (left y-axis). The most associated SNP and HLA allele in each step of the stepwise conditional logistic regression analysis are indicated by squares. Recombination rates from the HapMap CEU are depicted in blue (right y-axis). Genomic positions on the x-axis are based on the NCBI Build 36 (hg 18) assembly. Mb, megabase pairs.

We next performed a conditional logistic regression analysis, using the genotype of the extended HLA-B*0801-DRB1*0301-DQB1*02 as a covariate. The imputed HLA-DRB1*0701 allele was found to be the most significantly associated marker (combined *P_cond_* = 1.77×10^−22^; Figure 3).

In contrast to DRB1*0301-containing haplotypes, haplotypes linked to the DRB1*0701 allele have a high rate of historical recombination (Figure 2). Although, DRB1*0701 is found frequently within the HLA-B*13-DRB1*0701-DQB1*02, B*44-DRB1*0701-DQB1*02 and B*57-DRB1*0701-DQB1*0303 extended haplotypes, the DRB1*0701 allele can also be found on other haplotypes, in association with different HLA Class I and Class II alleles (Figure 2). However, all DRB1*07 haplotypes carrying the DQB1*02 allele were found to be similarly associated with increased risk to IgAD (combined *P* = 6.35×10^−17^; OR = 2.23, 95% CI 1.83–2.72; Table 1). Conversely, we found no strong evidence for the association of recombinant (non-DQB1*02) DRB1*07 haplotypes with IgAD (combined *P* = 0.027; OR = 1.34, 95% CI 0.96–1.86; Table 1). Further supporting this observation, we have over 80% power to detect the association of recombinant (non-DQB1*02) DRB1*07 haplotypes at α = 5×10^−8^ (Table S2). These data point to an important role of the DQB1*0202 allele, or a region in tight linkage with DQB1*0202, in the association of the DRB1*0701-DQB1*02 haplotypes with IgAD. In summary, the data suggest that DRB1*0301 and DRB*0701 haplotypes independently contribute to risk of IgAD, and the signal at DQB1*02 is essentially the sum of these independent effects.

### The HLA-B*1402-DRB1*0102-DQB1*05 haplotype is an independent risk factor for IgAD

After further conditioning on both the HLA-B*0801-DRB1*03-DQB1*02 and –DRB1*0701-DQB1*02 haplotypes, the most significant residual HLA association signal corresponded to the HLA-DRB1*0102 allele (*P*_cond_ = 2.95×10^−19^; Figure 3). Association of the DRB1*01 allele with IgAD has been well documented, and contains both the more common DRB1*0101 and the less common DRB1*0102 subtypes. Importantly, we found that the association with IgAD was exclusively contributed by haplotypes containing the DRB1*0102 allele, which showed association with risk in all 3 independent European populations (combined *P* = 5.86×10^−17^; OR = 4.28, 95% CI 2.92–6.26; Table 1). Conversely, we found essentially no evidence for the association of the HLA-DRB1*0101-DQB1*05 haplotype with IgAD (combined *P* = 0.038; OR = 1.17, 95% CI 0.98–1.39; Table 1).

Haplotype analysis showed that the DRB1*0102 is usually present on the extended B*1402-DRB1*0102-DQB1*0501 haplotype, which is a rare haplotype in Northern Europeans (0.2% allele frequency), but more common in the Spanish population (3% allele frequency; Table 1). Nevertheless, the DRB1*0102 allele was a strong risk factor in all 3 cohorts, and contributed the strongest individual effect of all the variants in the HLA region, with a combined OR = 4.59 (95% CI 2.89–7.30, Table 1). The recombination rate was modest across the extended B*1402-DRB1*0102-DQB1*05 haplotype. Of the 140 DRB1*0102-containing haplotypes, 47 (33.6%) were recombinant (non-B*1402; Figure 2). Despite the low number of recombinant haplotypes, the trend towards increased risk was maintained, even in the absence of the B*1402 allele (combined *P* = 3.62×10^−3^; OR = 3.22 95% CI 1.70–6.13; Table 1). In addition, in a single-locus association test, the HLA-DRB1*0102 allele was found to be more significantly associated with IgAD than the HLA-B*14 allele alone (combined *P* = 1.07×10^−12^; OR = 2.68; 95% CI 1.99–3.61; data not shown), suggesting that the causal allele on the extended B*1402-DRB1*0102-DQB1*05 is likely to be closer to the *HLA-DRB1* than to the *HLA-B* region.

### The HLA DRB1*1501-DQB1*06 haplotype confers protection from IgAD

To characterize additional independent loci, we next conditioned on the HLA-B*0801-DRB1*03-DQB1*02, -DRB1*0701-DQB1*02 and -DRB1*0102 alleles. The top residual association signal corresponded to the protective HLA-DRB1*15 allele (*P*_cond_ = 3.26×10^−18^; Figure 3). The protective effect of the HLA-DRB1*15 allele was evident in all 3 cohorts (combined *P* = 2.24×10^−35^; OR = 0.13; 95% CI 0.09–0.19; Table 1), and was observed for both DRB1*15 subtypes: the common DRB1*1501 allele, which was found to travel mostly on the extended B*0702-DRB1*1501-DQB1*06 haplotype, and the rare DRB1*1502 allele.

DRB1*1501 haplotypes showed a relatively high rate of historical recombination, and 51.2% excluded B*0702 (Figure 2). However, the recombinant DRB1*15 haplotypes were equally associated with protection from IgAD (combined *P* = 3.70×10^−18^; OR = 0.14, 95% CI 0.09–0.24; Table 1), suggesting that the causal variant associated with the DRB1*1501 signal is located within the linkage disequilibrium (LD) block containing *HLA-DRB1* and *HLA-DQB1* in the Class II region.

### Additional secondary association signals in the HLA region

We next performed further conditional logistic regression analysis, conditioning on all four association signals noted above: B*0801-DRB1*03-DQB1*02, -DRB1*0701-DQB1*02, DRB1*0102 and DRB1*1501. The association signal was significantly reduced, with only a few additional SNPs reaching the genome-wide association significance threshold (*P*<5×10^−8^; Figure S1). These SNPs mapped specifically to two discrete genomic regions: (i) the *BTNL2-HLA-DRA* locus on the telomeric end of the HLA class II region (rs743862; *P*_cond_ = 8.07×10^−14^; Figure S1); and (ii) the *HLA-DQB1* region (rs9275141; *P*_cond_ = 1.48×10^−11^; Figure S1), supporting the contribution of these two additional regions to risk for IgAD.

## Discussion

Markers in the HLA region represent the strongest genetic risk factor associated with IgAD. Nevertheless, the extensive conservation of the disease-associated haplotypes has hindered our ability to confidently map the causal variants. In this study, we performed the largest fine-mapping effort to date of the HLA locus in IgAD, using a high-density SNP panel to characterize the complex association at this locus, and to map the location of the independent susceptibility loci.

The primary association signal in the HLA region was found to be the HLA-DQB1*02 allele, including both the DQB1*0201 and the DQB1*0202 alleles, which are linked with the HLA-DRB1*03 and -DRB1*07 haplotypes respectively. Using a haplotype-based analysis, we found that the primary association signal was caused by the additive effect of two independent susceptibility alleles located on the extended HLA-B*08-DRB1*0301-DQB1*02 and DRB1*0701-DQB1*02 haplotypes. Importantly, by using a long-range haplotype phasing approach in a large sample size, we were able to demonstrate that recombinant (non-B*0801) DRB1*0301 haplotypes carrying the DQB1*0201 allele showed no evidence of association with disease. Despite the low historical recombination rate on DRB1*0301 haplotypes, this approach allowed us to compare a sufficiently large number of recombinant haplotypes to increase our confidence in these observations. Interestingly, the frequency of extended DRB1*0301 haplotypes was consistently increased in IgAD patients from all 3 independent populations, suggesting that, despite obvious population specific differences in the distribution of these haplotypes, the underlying association signal is identical in the different populations. Similarly, recombinant (non-DRB1*0301) B*0801 haplotypes, showed no evidence of association with IgAD, suggesting that the causal allele in the extended HLA-B*08-DRB1*0301-DQB1*02 is likely to be located in the telomeric end of the Class II region or in the Class III region.

To characterize further independent susceptibility loci, we next performed a stepwise conditional logistic regression analysis. Following conditioning on the primary HLA-B*08-DRB1*0301-DQB1*02 association signal, the most significantly associated HLA allele was found to be the DRB1*0701 allele, further supporting the presence of an independent risk allele on DRB1*07 haplotypes. Despite the high recombination rate, we found that DRB1*07 haplotypes carrying different Class I allele were all similarly associated with IgAD. This was in contrast to non-DQB1*0202 recombinant haplotypes, which showed no evidence for association. The approach used in this study to compare the association of the recombinant haplotypes in a large cohort to the respective extended risk haplotypes was validated by power calculations (summarized in Table S2) showing that the lack of association of the recombinant (non-B*0801) DRB1*0301, (non-DRB1*0301) B*0801 and (non-DQB1*02) DRB1*07 haplotypes are not likely to be negative results due to a lack of power in the study. Taken together, these data point to a causal role of the DQB1*0202 allele or another variant in tight LD with it in the *HLA-DQA1–DQB1* locus in the Class II region.

We therefore propose a model whereby the strong association observed with DQB1*02 allele results from the additive effect of two independent risk loci: the DQB1*0202 allele, or another marker in high LD and close proximity, traveling on HLA-DRB1*0701-DQB1*0202 haplotypes and an independent allele, most likely located within the border of the HLA Class III and Class II region, traveling specifically on the extended HLA-B*0801-DRB1*0301-DQB1*02 haplotype.

Conditioning on the HLA-B*0801-DRB1*0301-DQB1*02 and -DRB1*0701-DQB1*02 association signals, we characterized two additional secondary association signals in the HLA region. The HLA-DRB1*0102 allele was found to be a robust independent risk allele, while DRB1*1501 was strongly protective. The most significant secondary association signal was the DRB1*0102 allele traveling on the extended HLA-B*1402-DRB1*0102-DQB1*05 haplotype. The prevalence of the B*1402-DRB1*0102 haplotype showed marked population differences, and was more frequent in populations of Southern European ancestry, as evidenced by the 4.5% allele frequency in the Spanish controls, compared to only 0.3% and 0.4% in the Swedish and Finnish controls respectively. To have a better estimate of the DRB1*0102 allele frequency in Northern Europeans, we performed an extensive survey of the Swedish volunteer bone marrow donor registry (the Tobias registry). We identified 296 copies of the B*1402-DRB1*0102 haplotype in 23,610 healthy individuals that were surveyed, corresponding to a 0.6% allele frequency in the Swedish population. Despite the specific population differences, the DRB1*0102 association was consistent in all 3 independent cohorts and showed the highest OR of any HLA allele. Interestingly, the prevalence of IgAD in the Iranian population has been shown to be similar to that observed in European populations [30]. However, in Iranians the strongest HLA association with IgAD is linked to the B*14 and DRB1*01 alleles [31]. Taken together, these data clearly support the presence of a strong risk variant on the conserved HLA-B*1402-DRB1*0102-DQB1*05 haplotype. In fact, the effect size observed with this allele is consistent with the presence of a rare variant with strong penetrance on the HLA-B*1402-DRB1*0102-DQB1*05 background. Importantly, the lack of association with the more common DRB1*0101 subtype, which shares the association with the DQB1*05 allele, and the strong association with recombinant DRB1*0102 haplotypes, supports the location of the causal variant telomeric to the Class II region, most likely within the HLA Class III region. It is interesting to note that the 2 missense mutations that we have recently characterized in *MSH5*, L85F and P786S, located in the HLA Class III region, travel specifically on this extended haplotype [32]. It should be noted, however, that this hypothesis was not supported by a recent study by Pozo *et al*, suggesting that recombinant (non-DRB1*0102) haplotypes carrying the L85F variant did not show evidence of association with IgAD [33]. Nevertheless, the conservation and low frequency of recombinant HLA-B*1402-DRB1*0102-DQB1*05 haplotypes in European populations, warrant further analyses and a larger sample size to confirm whether these missense mutations are the causal variants for IgAD in this haplotype.

Another independent association signal identified was the protective effect conferred by the DRB1*1501 allele. The association of DRB1*1501 with disease protection has been well documented, and further supports the important role of variants in the HLA Class II region to the complex association signal observed on the HLA locus. In fact, although the recombination rate in the DRB1*1501 haplotypes was elevated, all haplotypes sharing the HLA Class II fragment containing the DRB1*1501 allele conferred similar protection from IgAD. These data support the location of a protective allele mapping specifically to the LD block containing the HLA-DRB1 and HLA-DQB1 genes, and are consistent with the previous hypothesis that the protective effect is due to the presence of a negatively charged aspartic acid in position 57 of the HLA-DQβ chain in DRB1*1501 haplotypes. In contrast, in risk haplotypes, the aspartic acid residue is replaced by a neutral alanine [12].

Of interest, the association with the HLA locus in IgAD shares some striking similarities with the association in type 1 diabetes (T1D), where the DRB1*0301 allele is a strong risk factor and the DRB1*1501 allele confers protection against the disease [34], [35]. Similarly we have recently characterized the association between IgAD and two novel non-HLA loci, *IFIH1* and *CLEC16A* [24], which are also known to be associated with T1D [36], suggesting a shared genetic predisposition to both diseases. Despite these similarities, there are some notable disease-specific differences in the association with the HLA between the two conditions: i) the DRB1*04 allele, one of the strongest risk factor in T1D, is not associated with IgAD; ii) unlike T1D, the DRB1*0301 association with IgAD is restricted to the extended B*0801-DRB1*0301-DQB1*02 haplotype; iii) the DRB1*0701 allele, which is associated with increased risk of IgAD is protective in T1D, while the DRB1*0102 allele shows no evidence for association with T1D [34], [35]. These data suggest that the association with the HLA locus is the result of multiple independent effects, some of which are shared between different diseases. The characterization of the association signal in the HLA region in different diseases may, therefore, contribute important insights into the location of shared genetic effects, and may help in the identification of the causal variants in this genomic region.

In addition to the association of the extended HLA haplotypes, we found further evidence for association of a few additional independent markers in the HLA locus. Most notable were the associations with SNPs in the *BTNL2-HLA-DRA* locus on the telomeric end of the Class II region and in the *HLA-DQB1* region. Taken together, these data confirm that IgAD susceptibility is the result of multilocus effects that span the entire HLA region.

**Figure 4:**
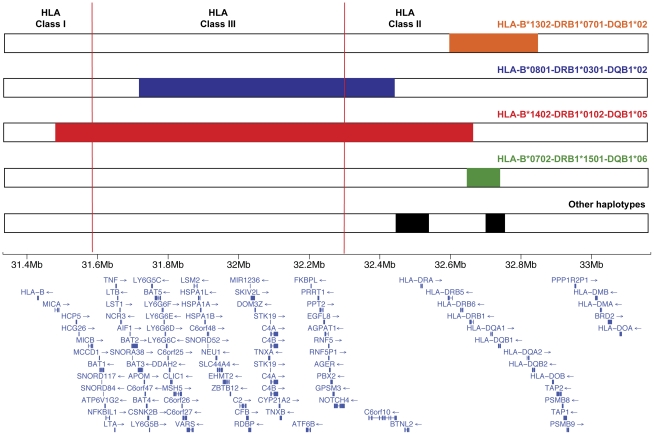
Summary of most significant independent association signals in the HLA locus. For each conserved haplotype background the critical region where the putative causal variant is more likely to be located is highlighted. Genomic positions on the x-axis are based on the NCBI Build 36 (hg 18) assembly. Mb, megabase pairs.

In summary, we have mapped the primary susceptibility locus to the HLA Class II region and found evidence for the association of additional independent loci in Class III and Class I regions. Importantly, the fine-mapping strategy provided a better resolution of the individual haplotype background and their specific contribution to disease susceptibility. A summary of the putative causal alleles and their respective location on the specific haplotype background is depicted in Figure 4. These data are consistent with a previous report from de la Concha *et al.* that used a similar haplotype-based analysis to determine the location of the IgAD causal variants in the HLA locus in the Spanish population [37]. Here, we build on these results and extend the findings to other European populations using a high-density SNP mapping approach to reconstruct the individual HLA haplotypes with high confidence. Taken together, these studies indicate that although the HLA locus shows strong population differences, the haplotype-specific genetic association signals are similar in the different cohorts, suggesting that conserved causal variants, present in ancestral haplotypes confer similar genetic predisposition to disease independently of the ethnic background. This information will also help generate better models to test the putative interaction of specific HLA variants with the non-HLA risk loci identified through the genome-wide association effort. In addition, the characterization of the role of each specific haplotype to disease susceptibility may provide important information about the precise location of the functional variant(s) within the haplotype. These data can provide a rationale to prioritize the specific conserved haplotype segments that should be targeted for future sequencing efforts. With the advent of more cost-effective sequencing technologies, the complete re-sequencing of large haplotype segments in a large targeted sample will become feasible, and may be the only definitive approach to identify the functional HLA variants present on these extremely conserved extended haplotypes.

## Methods

### Subjects

A total of 861 IgAD patients were enrolled from 4 different centers across Europe: 418 were recruited at the Karolinska Institutet, Karolinska University Hospital Huddinge; 34 were recruited at the Landspitali – University Hospital, Reykjavik; 280 were recruited at the Hospital Universitario La Paz in Madrid; and 129 were recruited from the Finnish Red Cross Blood Service in Helsinki. The diagnosis of IgAD was obtained according to accepted guidelines, with serum IgA levels below the detection threshold (IgA<0.07 g/l), as measured by nephelometry in multiple independent blood samples [5], [38]. 2,184 geographically matched control samples were obtained from 3 independent sources: 1,115 control samples from the Epidemiological Investigation of Rheumatoid Arthritis (EIRA) Swedish inception cohort; 373 samples collected at Hospital Clínico San Carlos in Madrid; and 693 samples from the Nordic Centre of Excellence in Disease Genetics consortium (NCoEDG; Finnish controls). All DNA samples were collected after approval from the relevant research ethics and committees.

### Genotyping and control quality

IgAD patients and the Spanish controls were genotyped at the Feinstein Institute, New York, and the Swedish controls were genotyped at The Genome Institute of Singapore. All genotyping was performed using the Illumina BeadChip technology, on either the HumanHap 300 or Human 610-quad chips. Genotyping data for the Finnish controls was obtained from the NCoEDG, and was generated using the Illumina CNV370 platform.

To assure high quality data on the final analysis, we used stringent quality control measures, as described previously [24]. The final number of individuals passing all the quality control steps in the 3 independent case-controls series were as follows: Sweden/Iceland: 430 cases and 1,090 controls; Spain: 256 cases and 322 controls; and Finland: 86 cases and 564 controls. We observed minimal inflation of the median χ^2^ statistic in the 3 populations (Sweden/Iceland: λ_GC_ = 1.05; Spain: λ_GC_ = 1.05; Finland: λ_GC_ = 1.04), thus ruling out potential population stratification issues on the different case-control series. Fine-mapping of the HLA region was performed by extracting the 1,686 SNPs spanning 10 Mb of chromosome 6 (25–35 Mb) that passed all quality control steps.

### SNP imputation

SNP imputation of the HapMap2 (release# 24) reference dataset was performed using IMPUTE [39], with default settings. Imputation was performed using only the SNPs that passed quality control and were genotyped in all 3 case-control series. To ensure that only SNPs imputed with high confidence were included in the final analysis, we only tested the association of SNPs reaching an imputation score (proper_info statistic) >0.8 in each case-control series.

### HLA typing

The IgAD patients from Sweden, Iceland and Finland were genotyped at the *HLA-B, -DRB1* and *-DQB1* loci using PCR-SSP [40] employing the *HLA-B* low resolution and the *HLA-DQ*, *-DR* SSP Combi Tray kits from Olerup SSP AB, Saltsjöbaden, Sweden.

In the Spanish samples, *HLA-B* was typed using the Low Resolution SSP Typing kit by Biosynthesis (Lewisville, TX). *HLA-DRB1* typing (and subtyping) and *-DQB1* typing were conducted by PCR amplification and hybridization with allele-specific oligonucleotides [41].

### Imputation of genotypes at the common HLA-B, -DRB1, and -DQB1 genes

The SNP data was used to impute the common *HLA-B*, -*DRB1* and -*DQB1* alleles in all the samples studied. The use of SNP data has been previously shown to be useful in inferring HLA alleles by taking advantage of the strong LD structure in the region. de Bakker *et al* showed that using genotype information from just 1 to 3 neighboring SNPs (tagging SNPs), they were able to accurately infer the genotypes of the most common HLA alleles [42]. Nevertheless, the accuracy of this tagging SNP approach is affected by the variability of most classical HLA alleles, and does not provide complete information about the haplotype background surrounding the HLA alleles and about the occurrence of internal recombination events within the haplotypes. More recently, a different approach has been proposed, using multi-maker SNP data across the entire HLA region to infer the haplotype context surrounding the alleles, and to provide a more accurate estimation of the specific HLA alleles [43].

The SNP selection strategy employed in this study for haplotype phasing included the initial selection of 11 HLA-tagging SNPs that were found to be the most informative for the imputation of the more common *HLA-B*, *-DRB1* and *-DQB1* alleles, using the tagger algorithm implemented in PLINK [44]. Then, we supplemented the set of tagging SNPs with a second set of 38 SNPs distributed uniformly across the classical HLA region. Importantly, to capture the full diversity of the haplotype structure and to map the individual recombination events, SNP selection was restricted to common and independent (low pairwise r^2^) SNPs. Given the lack of accuracy of the tagging approach to infer most *HLA-B* alleles, we included a higher density of SNPs for the HLA Class I region. The genotype information from the final set of 49 SNPs (listed in Table S3) was used to reconstruct the individual long-range haplotypes, using PHASE v2.1 [45], with the standard pre-defined parameters. Missing genotypes from the final set of 49 SNPs were also imputed during the phasing step.

The imputation of the common HLA alleles was then performed by aligning the phased haplotypes and by defining, visually, the longest segment of consecutive SNPs surrounding the tagging SNP that uniquely identifies each common HLA allele. This strategy was possible because we had access to a large number of samples that were HLA typed at the HLA-B, HLA-DRB1 and HLA-DQB1 genes. In fact, 2 or 4-digit *HLA-B* typing data was available for 410 IgAD patients, while *HLA-DRB1* and *–DQB1* data was available from 534 and 638 IgAD patients respectively (Table S4).

To test the performance of the method, the sensitivity, specificity and positive predictive value (PPV) were calculated on both the training set and on an additional validation set of 79 IgAD patients that were used to determine the allele-specific haplotype segments. Estimation of the sensitivity, specificity and PPV of the imputed alleles in the validation set was very high (Table S5). The only notable exceptions were the B*0702 allele, which was found to be rare in the validation cohort, and the DQB1*03 allele (Table S5). Given the computing-intensive nature of the haplotype phasing, the number of SNPs used for this step was limited, and, most likely, insufficient to fully characterize rare HLA alleles. However, it was sufficient to accurately depict the haplotype background of the common HLA alleles that have previously been associated with risk or protection for IgAD.

### Statistical analyses

#### Allele-based association tests

Allele-based association tests were performed on all SNPs and imputed HLA alleles. Association of all variants to disease was calculated using a 1-degree of freedom χ^2^ test, comparing the minor allele frequency in cases and controls in each population. Haplotype association tests were performed using a similar strategy, by comparing the frequency of the selected haplotype against all other haplotypes combined in cases and controls from each cohort. For imputed SNPs, allele-based association tests were performed using SNPTEST (https://mathgen.stats.ox.ac.uk/genetics_software/snptest/snptest.html). The association statistics were corrected taking into account the accuracy of the imputation method for each SNP. All allele-based association tests were performed assuming an additive risk model.

Combined *P* values, were calculated performing a meta-analysis of the 3 independent case-control series, using a weighted z-score method implemented in the METAL software package (http://www.sph.umich.edu/csg/abecasis/metal/). In each cohort, *P* values were converted to z-scores taking into account the direction of the effect relative to an arbitrary allele. A weighted sum of z-scores was calculated by dividing the individual z-scores by the square root of the sample size of each cohort and then dividing the sum by the square root of the total sample size. The reported combined *P* values were obtained by converting the meta-analysis z-scores into two-tailed *P* values.

To calculate combined odds ratios, we used the Cochran-Mantel-Haenszel method implemented in PLINK [44]. All odds ratios were calculated relative to the minor allele on the Swedish/Icelandic population. The genomic inflation factor was estimated in each cohort from the median χ^2^ statistic after excluding all SNPs from the HLA region.

#### Conditional regression analyses

To characterize all independent association signals from the known HLA alleles, a stepwise forward conditional logistic regression analysis was performed on the 3 independent cohorts, controlling for the genotypes of the most associated HLA alleles or haplotypes. The analysis represents a regression of disease status on each genotyped test SNP and imputed HLA allele, including the genotype at the most associated HLA allele (coded as 0,1 and 2 according to the number of minor alleles) as covariates. In each step the genotype of the top residual associated HLA allele was added as an additional covariate to the model and the association was calculated in all remaining test SNPs and imputed HLA alleles. The process was then repeated iteratively, until no additional HLA allele reached genome-wide association. All conditional analyses were performed using PLINK [44]. Conditional analyses were performed in each case-control series independently, and combined conditional *P* values were calculated using a meta-analysis, as described above.

#### Power calculations

The power to detect the association of the observed recombinant haplotypes with IgAD was calculated using the CaTS power calculator for association studies (http://www.sph.umich.edu/csg/abecasis/CaTS/), assuming a multiplicative disease model, a disease incidence of 1∶500 and a single-stage study design. To determine the effect size of the extended risk haplotypes, we first computed the genotype relative risk (GRR) corresponding to the observed frequencies of the risk allele in IgAD patients and controls. The same GRR was then applied to the respective recombinant haplotypes to calculate the power to detect disease association in our combined sample of 772 IgAD patients and 1,976 controls at the significance level (α) of 0.05, 0.01, 5×10^−5^ and 5×10^−8^.

## Supporting Information

Figure S1 Residual association signal in the HLA locus following conditioning on all major association signals. Combined *P* values are shown for all SNPs and imputed HLA alleles, following conditional logistic regression analysis, using the genotypes at the HLA-B*0801-DRB1*0301-DQB1*02, -DRB1*0701-DQB1*02, -DRB1*0102 and -DRB1*15 alleles as covariates. Association results are represented as the −log_10_ of the combined *P* values (left y-axis), and the most associated SNP (rs743862) and imputed HLA allele (B*1501) are marked by squares. Recombination rates from the HapMap CEU are depicted in blue (right y-axis). Genomic positions on the x-axis are based on the NCBI Build 36 (hg 18) assembly. The horizontal red line represents the genome-wide significance threshold of *P* = 5×10^−8^. Mb, megabase pairs.
(TIF)

Table S1 Distribution of HLA-DRB1*0301 haplotypes in the 3 cases-control series. Shown are summary statistics for the distribution of extended and recombinant (non-B*0801) HLA-DRB1*0301 haplotypes in the 3 case-control series. *P* values were calculated using a two-tailed Fisher's exact probability test comparing the frequency of recombinant DRB1*0301 haplotypes in cases and controls. Odds ratios were calculated relative to the frequency of extended HLA-B*0801-DRB1*0301-DQB1*02 haplotypes. Combined *P* value and odds ratio were calculated using a Cochran-Mantel-Haenszel test. ^a^ Extended DR3 represent all extended HLA-B*0801-DRB1*0301-DQB1*02 haplotypes; ^b^ Recombinant DR3 represent all recombinant (non-B*0801) DRB1*0301 haplotypes. N, number of haplotypes; OR, odds ratio.
(XLS)

Table S2 Power to detect the association of recombinant haplotypes with IgAD. Power to detect the association of the observed recombinant haplotypes with IgAD in our combined cohort of 772 patients and 1,976 controls. Power calculations were performed assuming a multiplicative model and a disease incidence of 1∶500. The relative risk of each recombinant haplotype was estimated to be identical to the relative risk observed for the respective extended risk haplotype. GRR, genotype relative risk.
(XLS)

Table S3 Final set of SNPs used for the reconstruction of the extended haplotypes. List of SNPs in the HLA locus selected to reconstruct the extended haplotypes. Chromosomal positions are based on the NCBI Build 36 (hg 18) assembly.
(XLS)

Table S4 HLA typing information in IgAD patients. Shown is the number of IgAD patients from each case-control series where 2-digit or 4-digit HLA typing information for the *HLA-B*, *-DRB1* and *-DQB1* alleles was available. N, Number of patients included in the study after applying all quality control filters.
(XLS)

Table S5 Performance of the HLA imputation method on the training and validation sets. Performance of the imputation method for estimating the *HLA-B*, -*DRB1* and -*DQB1* alleles in this study. Accuracy of the method was estimated by calculating the sensitivity, specificity and positive predictive value (PPV) in the training set (consisting of 410, 534 and 638 individuals with known *HLA-B*, -*DRB1* and -*DQB1* typing information, respectively) and an independent validation set (consisting of 79 individuals).
(XLS)
