# Supplementary figures and images for: High-Density SNP Mapping of the HLA Region Identifies Multiple Independent Susceptibility Loci Associated with Selective IgA Deficiency
Source: PLoS Genet. 2012 Jan 26;8(1):e1002476. doi: 10.1371/journal.pgen.1002476 (PMC3266887; doi:10.1371/journal.pgen.1002476)

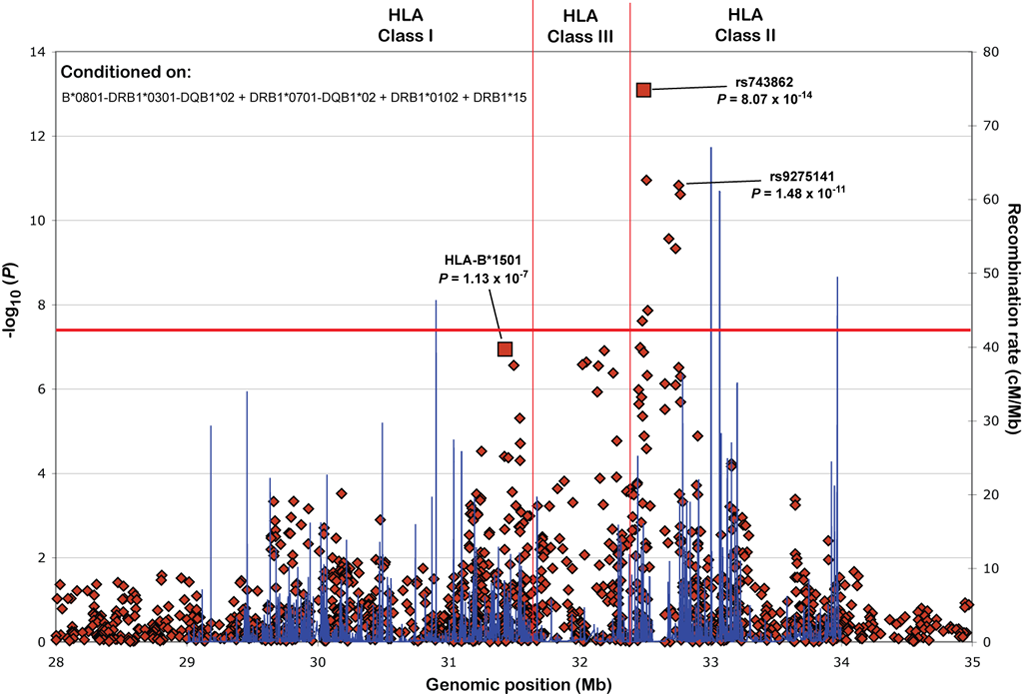

Supplement: Figure S1 — Residual association signal in the HLA locus following conditioning on all major association signals. Combined P values are shown for all SNPs and imputed HLA alleles, following conditional logistic regression analysis, using the genotypes at the HLA-B*0801-DRB1*0301-DQB1*02, -DRB1*0701-DQB1*02, -DRB1*0102 and -DRB1*15 alleles as covariates. Association results are represented as the −log10 of the combined P values (left y-axis), and the most associated SNP (rs743862) and imputed HLA allele (B*1501) are marked by squares. Recombination rates from the HapMap CEU are depicted in blue (right y-axis). Genomic positions on the x-axis are based on the NCBI Build 36 (hg 18) assembly. The horizontal red line represents the genome-wide significance threshold of P = 5×10^−8^. Mb, megabase pairs. (TIF) [file pgen.1002476.s001.tif]
